# Development of All India Institute of Medical Sciences-Modified International Clinical Epidemiology Network Diagnostic Instrument for Neuromotor Impairments in Children Aged 1 Month to 18 Years

**DOI:** 10.3389/fpubh.2017.00313

**Published:** 2017-11-21

**Authors:** Sheffali Gulati, Harsh Patel, Biswaroop Chakrabarty, Rachana Dubey, N. K. Arora, R. M. Pandey, Vinod K. Paul, Konanki Ramesh, Vyshakh Anand, Ankit Meena

**Affiliations:** ^1^Child Neurology Division, Department of Pediatrics, All India Institute of Medical Sciences, New Delhi, India; ^2^INCLEN Trust, New Delhi, India; ^3^Department of Biostatistics, All India Institute of Medical Sciences, New Delhi, India; ^4^Department of Pediatrics, All India Institute of Medical Sciences, New Delhi, India; ^5^Rainbow Children Hospital, Hyderabad, India

**Keywords:** All India Institute of Medical Sciences, International Clinical Epidemiology Network, INCLEN diagnostic tool, neuromotor impairment, tool

## Abstract

**Introduction:**

There is shortage of specialists for the diagnosis of children with neuromotor impairments (NMIs), especially in resource limited settings. Existing International Clinical Epidemiology Network (INCLEN) instrument for diagnosing NMI have been validated for children aged 2–9 years. The current study modified the same including wider symptomatology and age group (1 month to 18 years).

**Methods:**

The Modified INCLEN diagnostic tool (INDT) was developed by a team of experts by modifying the existing tool to widen the age range (1 month to 18 years) and include broader symptomatology (inclusion of milestones from the first 2 years of life and better elucidation of cerebellar and extrapyramidal features) in a tertiary care teaching hospital of North India between January and April 2015. A trained medical graduate applied the candidate tool, which was followed by gold standard evaluation by a Pediatric Neurologist (both blinded to each other).

**Results:**

A total of 197 children (102 with NMI and 95 without NMI) were enrolled for the study. The sensitivity, specificity, positive and negative predictive values, positive and negative likelihood ratio of the modified NMI tool were 90.4% (82.6–95.5), 95.5% (88.7–98.7), 95.5% (88.9–98.7), 90.3% (82.4–95.5), 19.9 (12.1–32.6), and 0.13 (0.08–0.12), respectively.

**Conclusion:**

The All India Institute of Medical Sciences modified INDT NMI tool is a simple and structured instrument covering a wider symptomatology in the 1 month to 18 years age group with acceptable diagnostic accuracy.

## What This Paper ADDS?

In resource constrained settings like India, there is a dearth of specialists who can appropriately manage children with neuromotor impairments (NMIs). To empower primary care physicians to diagnose, manage, and refer these children correctly, International Clinical Epidemiology Network (INCLEN) developed and validated the INCLEN Diagnostic tool (INDT) for NMI in 2- to 9-year-old children with good diagnostic properties. The current study has modified this tool including wider age range (1 month to 18 years) and symptomatology. The latter includes elucidation of salient milestones in the first 2 years of life and a more comprehensive description of movement disorders. This will help in identification of disorders, which are fatal early in life and genetic counseling is of paramount importance and also those conditions in which early appropriate intervention can have optimal long-term outcome.

## Introduction

Neuromotor impairments include cerebral palsy (CP), progressive acquired or inherited neuromuscular disorders (NMDs), and other NMI (not satisfying the definition of either CP or NMD), which are a continuum of disorders that affect body functions, activities, and quality of life (1). The World Health Organization (WHO) report showed prevalence of neurodevelopmental disorders (NDDs) to the tune of 1–20% globally (2). The NMIs are among the most common NDDs affecting children and contribute to significant morbidity (3). The commonest form of NMI is CP, with a prevalence of 1.2–2.5 per 1,000 live births (4). There is variability seen in reported prevalence, which is partly attributed to lack of validated operational identification measures (5). Although there are many tools for classifications into subtypes based on topography and severity of impairment and disability, there is a dearth of structured, validated instruments for diagnosis of NMIs in children, which can be used by primary or secondary level health-care professionals. Hence, there is need to develop valid and socio-culturally appropriate diagnostic instruments, particularly in resource constrained settings, in order to enable primary and secondary level health-care professionals (without formal training in pediatric neurology) to diagnose NMIs with good accuracy. Toward this, the INCLEN developed and validated INCLEN Diagnostic tool for NMIs (INDT-NMI) between 2 and 9 years of age (1). The current study was planned to develop a modified version of INDT-NMI tool to cover wider range of symptomatology and age range (1 month to 18 years) compared to the first tool. This would facilitate early institution of appropriate diagnostic, treatment, and rehabilitation measures in this group of children.

## Methodology

The study was conducted at a tertiary care teaching hospital in northern India from January to April 2015. This center receives referred cases for complex medical problems as well as simple ailments seen at primary care level from entire India and neighboring middle and southeast Asia. Children aged 1 month to 18 years, of either gender, attending the Pediatric outpatient clinics were eligible for inclusion in the study. Children who had poor general condition requiring admission (viz., respiratory distress requiring supplemental oxygen, altered sensorium, peripheral circulatory collapse, suspected sepsis), those who were not accompanied by a primary caregiver and when care provider refused consent to participate were excluded from the study. The study was done in two phases. In the first phase, the All India Institute of Medical Sciences (AIIMS) modified version of INCLEN Diagnostic Tool for Neuromotor impairments (INDT-NMI) diagnostic tool for children was developed and the second step involved internal validation of the same. Ethical approval was obtained from Institute Ethics Committee.

### First Step: Development of the Tool

The AIIMS Modified INDT-NMI tool was developed as a modification of existing validated INDT for NMIs in children aged 2–9 years with the aim to cover a wider age group from 1 month to 18 years and a wider symptomatology. New questions were developed describing important developmental motor milestones for less than 2 years. Old questions were reframed and new questions were added to elicit extrapyramidal and cerebellar disorders with more accuracy. Diagnosis of CP using AIIMS modified INDT-NMI tool is based on the definition proposed by the Executive Committee on Definition and Classification of Cerebral palsy (6) and consensus clinical criteria for diagnosing CP and NMIs by the Technical Advisory Group consisting of pediatricians, pediatric neurologists, developmental pediatricians, child psychiatrists, pediatric clinical psychologists, special educators, and specialist nurses.

Questions of the tool eliciting information from parents were translated into Hindi and back translated to English as per protocol of the study. The modified tool in English has been represented in supplement. The Hindi tool was pretested in 10 children. A team of pediatric neurologists (two) with at least 3 years experience in the diagnosis and management of children with NMIs, MBBS graduate physicians (two), and one study coordinator participated in the study.

### Step 2: Internal Validation of AIIMS Modified INDT-NMI Tool

#### Sampling Method and Enrollment

Enrollment was done by the method of systematic random sampling. Computer generated two random numbers were provided to the study coordinator daily in a sealed envelope by the principal investigator. Starting point was decided by the first random number (between 1 and 9) and the second random number (between 5 and 15) determined the nth number (sample interval) to be sampled starting from the first random number. Every nth child from 1 month to 18 years of age visiting Pediatric outpatient’s clinic was assessed for eligibility and enrolled after obtaining written, informed consent from the primary caregiver until the final sample size was achieved based on the diagnosis by gold standard. If the caregiver was not willing to give consent or inclusion criterion was not fulfilled (n + 1)th child was enrolled.

After enrollment, subjects were first evaluated through candidate test (AIIMS modified INDT-NMI tool) administered by the Bachelor of Medicine and bachelor of surgery (MBBS) graduate physician. Administration of AIIMS modified INDT-NMI tool took approximately 20–25 min and the information was filled in predesigned structured *pro forma*, enclosed in a separate sealed opaque envelop bearing unique identification number, and handed over to the study coordinator. Later, the same subjects underwent gold standard evaluation (an expert team of two pediatric neurologists). Findings and final diagnosis by gold standard were handed over to the study coordinator in a structured format in opaque envelops. The sealed envelopes were opened at the end of day by the coordinator, who did not participate in the assessment of study subjects.

#### Sample Size

The previous study showed sensitivity of 75% and specificity of 86% for the first INCLEN diagnostic instrument for NMI in children aged 2–9 years (1). As the age group was widened and the tool modified in the current study, it was proposed to have atleast a sensitivity of 85% and a specificity of 90%. Hence with a relative precision of ±10% at 95% confidence level, it was required to enroll 51 children with NMI and 36 children with no NMI for AIIMS modified INDT-NMI tool for children aged 1 month to 18 years. To account for drop outs, it was planned to enroll at least 60 children with NMI and 45 children with no NMI.

#### Training for Administration of Candidate Tests

The MBBS graduate physicians were trained by pediatric neurologists in a comprehensive, hands on, structured workshop. The physicians were taught in detail about the administration of the diagnostic instrument (AIIMS modified INDT-NMI tool) using case vignettes and demonstration of methods of clinical examinations. Subsequently, the two candidates were evaluated for independent assessments on five patients each.

### Quality Assurance

**Figure 1:**
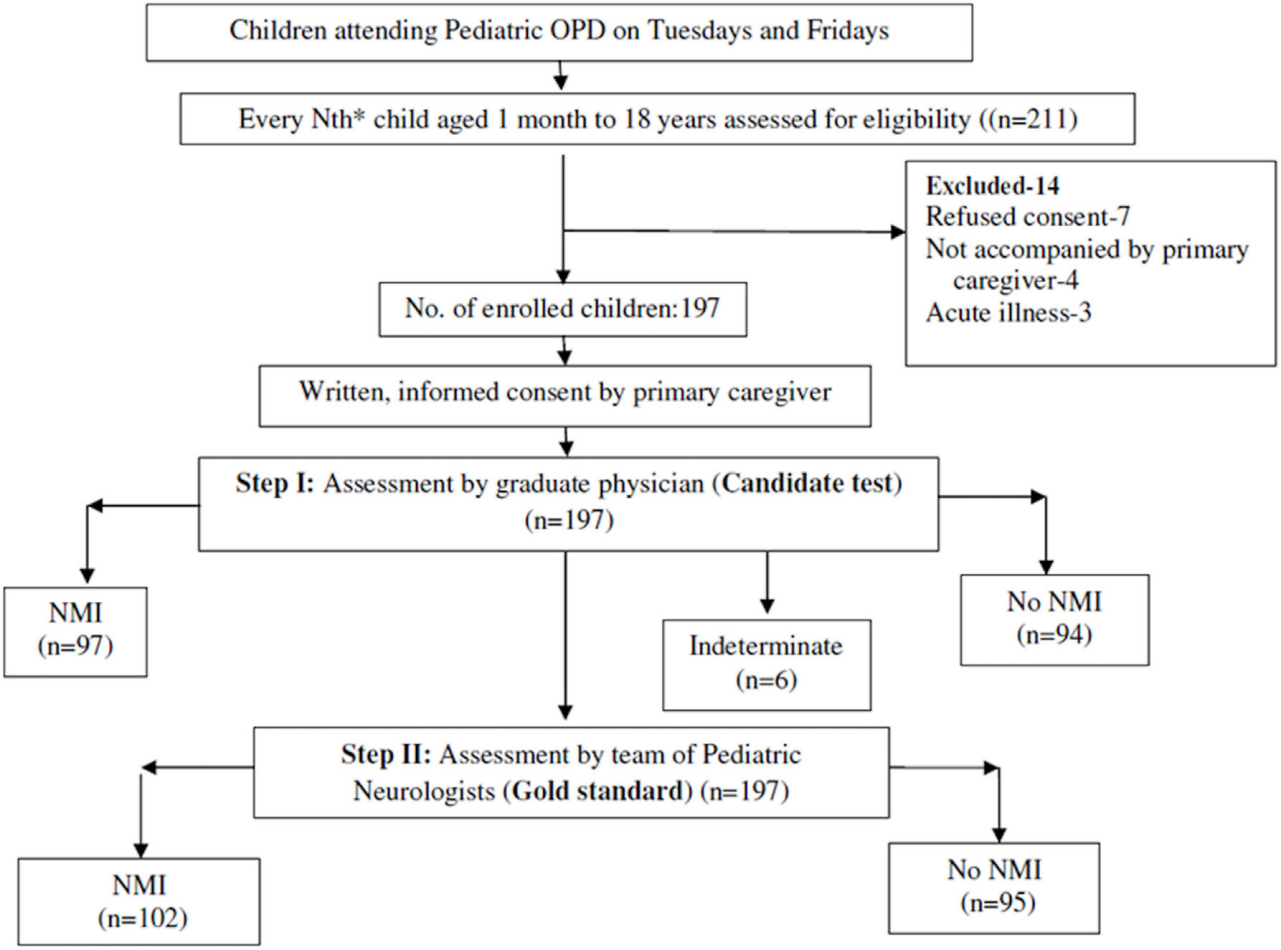
Flow of the study.

The team of pediatric neurologists (gold standard) was blinded to the assessment of the MBBS physician (candidate test) and vice versa. The study coordinator at the site assessed children attending the out-patient clinic for eligibility and enrolled them after taking written, informed consent from the primary caregiver, but did not take part in any of the assessments. Ethical approval was obtained from Institute Ethics Committee. The flow of the study has been depicted in Figure 1.

### Statistical Analysis

The data were analyzed using STATA v.11. The diagnostic properties of AIIMS modified INDT-NMI were ascertained in comparison with the assessments by the team of pediatric neurologists as gold standard. The sensitivity, specificity, positive and negative predictive values were expressed as percentages with 95% confidence interval (CI).

## Results

A total of 211 children were assessed for eligibility, of which, 197 children were enrolled for the study as 14 did not match the inclusion criteria. Of the 197 children, 102 were diagnosed as having “NMI” and 95 were diagnosed as “No NMI” by the gold standard (Figure 1*)*. Clinico-demographic characteristic are depicted in Table 1.

**Table 1:** Characteristics of study subjects with NMIs.

Total patients having NMIs	102
With other NDDs	45 (40%)
Without any NDDs	57 (60%)
Male	53 (52%)
Female	49 (48%)
Age (in months) median (IQR)	35 (18.88)
Mean ± SD	53.24 ± 43.18
Categories of NMI	
Cerebral palsy	61
Neuromuscular disorders	26
Other NMIs	15
Total no. children without NMI	95
With other NDDs	61 (65%)
Without other NDDs	34 (35%)

*NMI, neuromotor impairments; NDDs, neurodevelopmental disorders, which included epilepsy, intellectual disability/global developmental delay, autism, attention-deficit/hyperactivity disorder, hearing impairment, vision impairment and speech and language disorders; IQR, interquartile range*.

Of the 102 children with NMI as per gold standard assessment, 57 children had “NMI only,” and 45 children had “NMI with other NDDs.” Among the 102 children with NMI, 61 had CP, 25 had NMDs, and 15 had other NMIs. Among 95 children with “No NMI” as per gold standard assessment, 34 children were neurologically normal (“No NDD”), whereas 61 children had “Other NDDs,” i.e., NDDs other than NMI, including epilepsy, intellectual disability, autistic spectrum disorders, attention-deficit/hyperactivity disorder, hearing impairment, vision impairment, and speech and language disorders.

### Primary Outcome Measure

#### Diagnostic Accuracy of AIIMS-Modified INDT-NMI Tool for NMIs

The overall sensitivity and specificity of AIIMS modified INDT-EPI tool was 90.5% (95% CI: 82.6–95.53) and 95.5% (95% CI: 88.75–98.75), respectively. Positive and negative predictive value of the instrument was 95.5% (95% CI: 88.9–98.76) and 90.3% (82.42–95.5), respectively. Likelihood ratio of positive and negative results by instrument was 19.9 (12.16–32.55) and 0.13 (0.08–0.12), respectively (Table 2).

**Table 2:** Diagnostic utility of All India Institute of Medical Sciences modified International Clinical Epidemiology Network diagnostic tool NMI tool for NMIs.

		Gold standard		
		NMI	No NMI	Total
Test result	NMI	88	06	94
	No NMI	09	88	97
	Total	97	94	191

**Parameters**	**Values (CI)**			

Sensitivity %	90.43 (82.6–95.53)			
Specificity %	95.45 (88.75–98.75)			
PPV %	95.51 (88.9–98.76)			
NPV %	90.32 (82.42–95.5)			
LR+	19.9 (12.16–32.55)			
LR−	0.13 (0.08–0.12)			
Kappa	0.86 (0.71–1.0)			

*NMI, neuromotor impairment; PPV, positive predicitive value; NPV, negative predicitive value; LR, likelihood ratio; CI, confidence interval*.

In subgroup analysis for children aged 1 month to 2 years, this instrument showed sensitivity of 89.00 (75.95–96.29) and specificity of 88.50 (95% CI: 70.84–97.65), which is summarized in Table 3. Current instrument showed sensitivity of 93.7% (95% CI: 71.7–98.9) and specificity of 96.1% (95% CI: 81.1–99.3), respectively in children aged 9–18 years (Table 4). Tool administrator had put six cases under “indeterminate” category, out of which, five were diagnosed as NMI and one no NMI by gold standard. The rate of false positivity and false negativity of AIIMS Modified INDT-NMI tool were 9 and 6.3%, respectively.

**Table 3:** Diagnostic utility of All India Institute of Medical Sciences Modified International Clinical Epidemiology Network diagnostic tool-NMI tool in children aged 1 month to 2 years.

		Gold standard		
		NMI	No NMI	Total
Test result	NMI	40	03	43
	No NMI	05	21	26
	Total	45	24	69

**Parameters**		**Values (CI)**		

Sensitivity %		89.00 (75.95–96.29)		
Sensitivity %		88.50 (70.84–97.65)		
PPV %		93.02 (80.94–98.32)		
NPV %		80.77 (64.23–94.75)		
LR+		7.11 (3.7–13.75)		
LR−		0.13 (0.08–0.19)		
Kappa		0.75 (0.51–0.99)		

NMI, neuromotor impairment; PPV, positive predicitive value; NPV, negative predicitive value; LR, likelihood ratio; CI, confidence interval

**Table 4:** Diagnostic utility of All India Institute of Medical Sciences Modified International Clinical Epidemiology Network diagnostic tool-NMI tool in children aged 9–18 years.

		Gold standard		
		NMI	No NMI	Total
Test result	NMI	15	01	16
	No NMI	01	25	26
	Total	16	26	42

**Parameters**		**Values (CI)**		

Sensitivity%		93.7 (71.7–98.9)		
Specificity%		96.1 (81.1–99.3)		
PPV %		93.7 (71.6–98.9)		
NPV %		96.1 (81.1–99.3)		
LR+		24.3 (3.4–178.4)		
LR+		0.06 (0.009–0.5)		
Kappa		0.89 (0.59–1.2)		

*NMI, neuromotor impairment; PPV, positive predicitive value; NPV, negative predicitive value; LR, likelihood ratio; CI, confidence interval*.

## Discussion

The diagnosis of NMIs is essentially clinical. Although there are many tools for classification of diagnosed NMIs into subtypes based on topography and severity of impairment and disability, none of them are structured or validated to diagnose NMIs. An ideal diagnostic instrument should be acceptable, affordable, accurate, and reliable. The AIIMS modified INDT-NMI tool for children aged 1 month to 18 years has shown good diagnostic accuracy when applied by the MBBS physician. The acceptability and reliability of the current tool can be evaluated when it is externally validated in a community setting. There are no affordability issues as it is freely available.

There is unmet need for more number of Pediatric Neurologists in developed countries (7–9). Although there are no reliable estimates of the same in developing countries, this need is likely to be even higher. The current instrument is a “stand alone” instrument for whole pediatric age group, which can be used independently by a primary care physician to diagnose NMIs in children, which will facilitate early diagnosis, management, and appropriate referral.

The sensitivity of AIIMS modified INDT-NMI is 90.5% compared to 75.4% for the original INDT-NMI tool. This may be attributed to the fact that in the current study a wider age range was used and the descriptors were improved in the cerebellar and extrapyramidal parameters compared to the previous tool (Table 1). This study also showed a sensitivity and specificity of 92 and 95.5%, respectively in the 2–9 years age group compared to 75.4 and 86.8% of the original INDT NMI tool (1). Even in the age groups 9–18 years and 1 month to 2 years, the current modified tool showed sensitivity of 93.7 and 89%, respectively. Studies with larger sample size in each subgroup are desirable for conclusive validation in each age subgroup.

Cerebral palsy is essentially a clinical diagnosis. The WHO has proposed a two-staged approach involving initial community screening followed by diagnosis by an expert for characterizing NDDs in epidemiological studies. Some Indian studies have followed this protocol and used validated questionnaire to screen the populations for presence of various neurological disorders including NMIs (10–12). Studies have also used questionnaire based on motor developmental milestones to screen for delays and identify children with CP in a group of high-risk, prematurely born children below 2 years of age (13–15). In a multi-centric epidemiological study, an algorithm was developed based on selected components from standard neurological examination to identify CP in 2-year-old children who were born at extremely low gestational age (5). Recently, the Chinese version of INFANIB (Infant Neurological International Battery) for assessing infants with neuromotor abnormalities has been validated in primary care setting (16). The AIIMS modified INDT NMI tool is unique in the fact that it is an one step diagnostic tool for identifying children with the entire spectrum of NMI encompassing a very wide age range from 1 month to 18 years. Further, a robust study design, with an adequate sample size with a mix of normal children and children with other comorbid NDDs make this a good instrument for testing in future community-based epidemiological studies.

The American Academy of Pediatrics mentions the utility of developmental screening tools based on parent reporting like Ages and Stages Questionnaire (ASQ) and direct observation-based tools like Denver-II test. These are screening tools for developmental assessment and include evaluation of motor milestones as well (17). The Indian adaptation of ASQ has shown a sensitivity and specificity of 83.3 and 75.4%, respectively (18). In a cluster community study, The Denver-II battery showed a sensitivity of 83% with a modest specificity of 43% (19). However, these are developmental screening tools and can only predict children at risk of having developmental delay. The Alberta Infant Motor scale predicts gross motor skills of at risk infants and has a sensitivity and specificity of 80–90% across various age points in infancy (20). The INFANIB has a sensitivity and specificity of 75–90% across infancy (increasing values with advancing age) (16). In a community validation study, the Trivandrum Developmental Screening Chart for children up to 6 years of age demonstrated a sensitivity and specificity of 85 and 90%, respectively (21). The current tool has comparable and good psychometric properties across all age groups with respect to the other tools described above. The AIIMS modified INDT NMI tool is unique because it amalgamates motor developmental milestones with objective neurological clinical findings to not only identify children at risk of NMIs but also attempts to give them a label, which has significant diagnostic, prognostic, therapeutic, and rehabilitative implications. However, the current study has only developed the tool and conducted an internal validity. It now merits application at the community and primary health-care settings for external validation and wider application.

In low- and middle-income countries including India, up to 60% of children miss out the critical window of opportunity for early intervention for their optimum development due to low awareness among parents, inadequate sensitization of health-care providers, and lack of early intervention (22). The current tool by including milestones in the age group of 1 month to 2 years has tried to fill this gap to some extent. Early diagnosis of NMIs has two advantages. First, it will facilitate early institution of appropriate therapy and rehabilitation leading to each child achieving their maximal potential. Second, disorders that are rapidly progressive and are fatal in early life like spinal muscular atrophy type 1, early identification can help in appropriate genetic diagnosis and subsequent antenatal counseling. The commonest NMI is CP, upto 20% of which is comprised of dyskinetic and ataxic variants (23). The inclusion of questions addressing cerebellar and extrapyramidal features in the current tool would further help in identifying and managing these variants better.

The strengths of this study include an appropriate study design and an adequate sample size for development and modification of a diagnostic tool. There are certain limitations of the present study. The study set up is not truly representative of the community and the MBBS physicians (first year postgraduates at a tertiary care teaching hospital) do not truly represent primary care physicians in the country. However, the initial promising results call for application and validation at the primary and secondary health-care levels after health care providers are trained.

To conclude, AIIMS modified INDT-NMI tool is a diagnostic instrument with acceptable psychometric properties to diagnose different NMIs spanning age range from 1 month to 18 years. This instrument can be applicable for future epidemiological studies on NMIs. Correct diagnosis and prompt referral by primary health-care providers in resource limited settings can reduce the burden on tertiary health-care systems and allow appropriate, judicious, and cost-effective use of available resources.

## Author Contributions

SG, HP, and BC: conceptualization and conduct of the study, writing and reviewing manuscript. RD, VA, and AM: conduct of the study and reviewing manuscript. NA, RP, and VP: conceptualization and conduct of the study and reviewing manuscript. KR: conceptualization of the study and reviewing manuscript.

## Conflict of Interest Statement

The authors declare that the research was conducted in the absence of any commercial or financial relationships that could be construed as a potential conflict of interest.
